# Design and Photophysical Investigation of Schiff Base-Functionalized Pyrrolo[3,2-*c*]carbazoles

**DOI:** 10.1007/s10895-026-04792-7

**Published:** 2026-05-14

**Authors:** Gökhan Özbek, Esra Nur Kaya, Mehmet F. Saglam, Hakan Kandemir, Ibrahim F. Sengul

**Affiliations:** 1 Department of Chemistry, Faculty of Science, Gebze Technical University, Gebze, Kocaeli Turkey; 2 Department of Chemistry, Faculty of Art and Science, Tekirdag Namık Kemal University, Tekirdag, Turkey

**Keywords:** Pyrrolo[3,2-c]carbazole, Schiff base, Photophysical properties

## Abstract

Two pyrrolo[3,2-*c*]carbazole-based Schiff bases **5a** and **5b** were synthesized via condensation of pyrrolo[3,2-*c*]carbazole-2-carbohydrazide with benzaldehyde and *p*-bromobenzaldehyde. Their structures were confirmed by NMR, FT-IR, MALDI-TOF, and X-ray crystallography. UV–Vis spectra of the compounds showed bands at 300–380 nm, corresponding to π→π* and n→π* transitions, were showed minimal effects by solvent or concentration. Fluorescence studies revealed dual emission behavior, indicating the coexistence of multiple emissive states in the excited state, particularly in polar solvents. The fluorescence quantum yields of **5a** and **5b** were determined as 0.44 and 0.47, respectively. These results highlight the structural and photophysical versatility of pyrrolo[3,2-*c*]carbazole Schiff bases and demonstrate their potential for applications in fluorescent sensing and optoelectronic materials.

**Supplementary Information:**

The online version contains supplementary material available at 10.1007/s10895-026-04792-7.

## Introduction

**Fig. 1:**
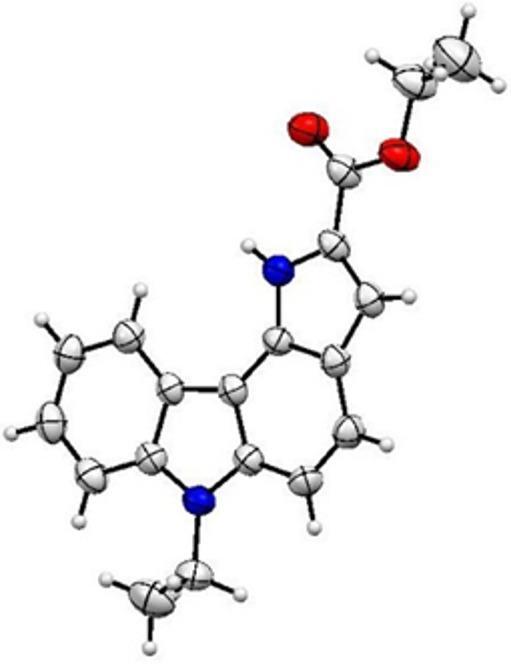
ORTEP diagram of compound **3** obtained from single-crystal X-ray diffraction analysis at 50% probability level (CCDC 2502062)

Carbazole is known as a tricyclic heteroaromatic compound consisting of pyrrole ring fused with two benzene rings. Carbazole and its derivatives have attracted considerable attention due to their excellent structural, electronic, and photophysical properties [1–2]. They act as efficient electron donors and exhibit strong absorption and emission characteristics, making them attractive for both biological and material applications. Carbazole based compounds have demonstrated diverse biological activities, including antimicrobial, anticancer, antioxidant, anti-inflammatory, and anti-diabetic [3]. Beyond the biological significance, carbazoles find applications in in materials science due to their semiconducting, photoconductive, and charge transport properties enable applications in organic light-emitting diodes (OLEDs), solar cells, nonlinear optical devices, and fluorescent chemical sensors [4–5]. Moreover, carbazoles exhibit chemical stability, thermal stability, and facile derivatization, enabling the fine-tuning of their optical and electronic properties [6]. Although carbazole derivatives occur in nature, they are also readily accessible through a wide range of synthetic methodologies [7]. From a synthetic perspective, the carbazole ring are easily functionalize, particularly at C3 and C6 positions and covalently linked to other molecules providing opportunity to construct new carbazole-based backbones with desire properties [8–10]. By exploiting these functionalization sites, it is thus possible to synthesize novel carbazole-based hybrid compounds with tailored properties. In this context, hybrid structures formed by the fusion of carbazole with other heterocyclic units have gained considerable attention due to their outstanding biological, photophysical, and photochemical properties [10–11]. Fused-ring carbazole derivatives have also played a significant role in the development of OLED materials, serving as traditional fluorophores, stable bipolar hosts for PhOLEDs, TADF emitters, and multiple-resonance (MR) emitters, highlighting their versatile photophysical and electronic properties [11]. Among carbazole fused systems, pyrrolo[3,2-*c*]carbazole, formed by fusion with a pyrrole ring, is an important class of annulated carbazoles (Fig. 1). Pyrrolo-carbazoles can be obtained from both natural products and laboratory synthesis. While pyrrolo-carbazoles can be obtained from both natural products and laboratory synthesis, most studies in the literature have primarily focused on their biological activities, with reports addressing their photophysical properties being relatively limited. For example, the photophysical behavior of pyrrolo[3,2-*c*]carbazole derivatives has been explored in only a single study, showing pronounced solvent dependence in both fluorescence intensity and absorption characteristics [12]. Several synthetic strategies, including Fischer indole synthesis, Hemetsberger indole synthesis, and transition metal-catalyzed reactions, have been employed to access diverse pyrrolo-carbazole frameworks [13–14].

Compounds containing the imine linkage *–N = CH-*, known as Schiff bases, are typically synthesized by the condensation reaction of primary amines and active carbonyl compounds, such as aldehydes or ketones, with the elimination of water [15]. Schiff bases have found extensive and important applications in both medicinal and supramolecular chemistry [15–16]. Additionally, organic compounds containing Schiff base units are excellent candidates for a diverse range of applications in photovoltaic materials, chemosensors, and organic light-emitting diodes (OLEDs), as they are capable of emitting light within specific wavelength ranges [17–19]. The presence of donor-acceptor pairs in Schiff base-functionalized pyrrolo[3,2-*c*]carbazole derivatives enables excited-state intramolecular proton transfer (ESIPT), a photophysical process in which a proton migrates within a molecule upon excitation, typically between amide or hydroxyl donor groups and carbonyl or imine acceptor groups. ESIPT gives rise to dual emission bands and large Stokes shifts, reflecting the coexistence of different tautomers in the excited state. This process is highly sensitive to molecular structure and solvent environment, making these derivatives promising candidates for tunable fluorescent materials and photophysical studies [20]. Encouraged by the various potential applications of pyrrolo-carbazoles and Schiff bases, we were interesting in developing a new class of pyrrolo[3,2-*c*]carbazole based Schiff bases and to investigate their photophysical properties.

## Results and Discussion

### Synthesis

**Scheme 1:**
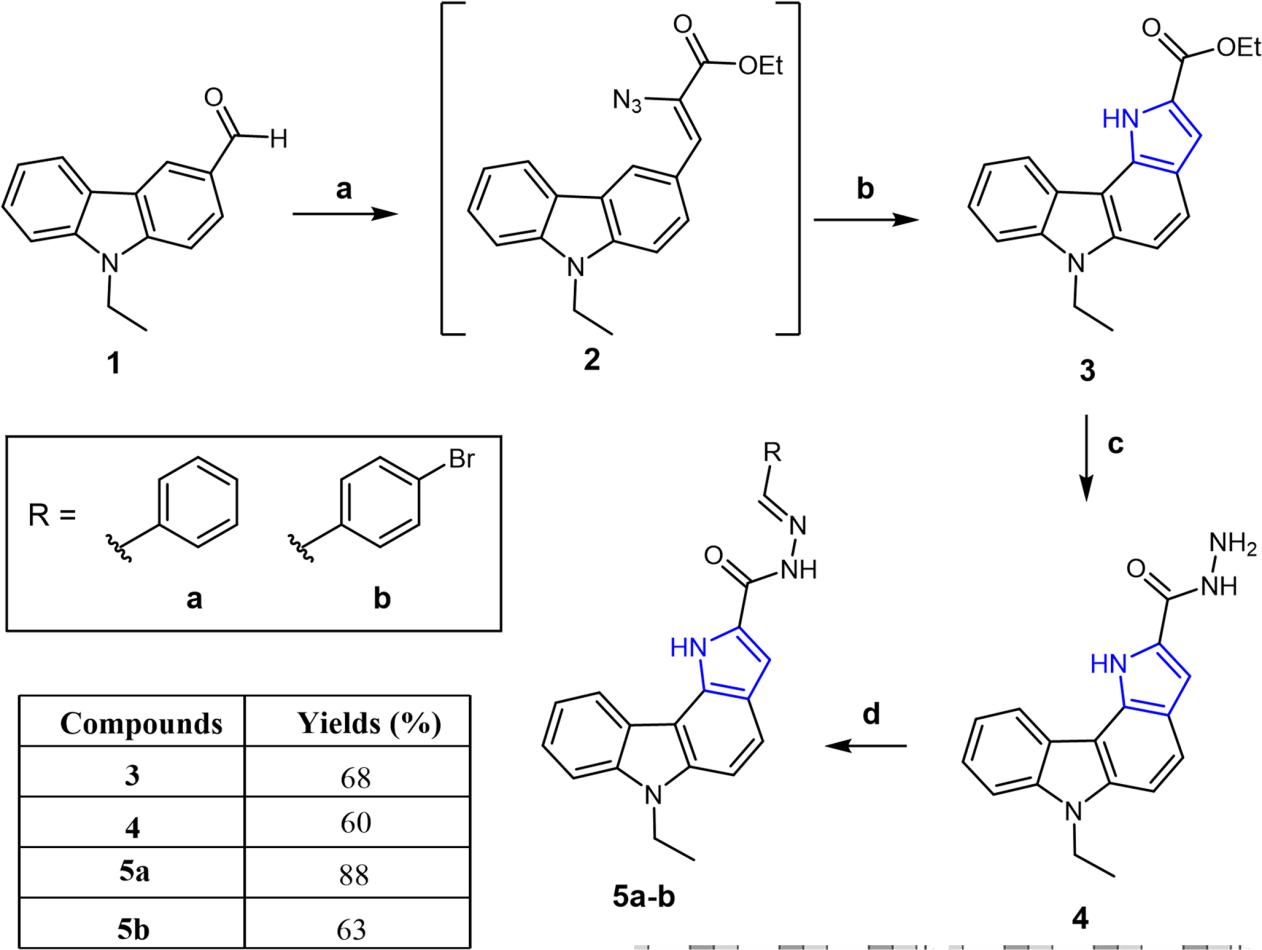
Reagents and conditions: **a**) N_3_CH_2_CO_2_Me, NaOMe, anhydrous EtOH, -15 °C, 4 h; **b**) 1,2-dichlorobenzene, reflux, 3 h; **c**) NH_2_NH_2_.H_2_O, EtOH, reflux, 20 h; **d**) ) RCHO, EtOH, AcOH

The aim of the synthetic part of this study is the preparation of novel pyrrolo[3,2-*c*]carbazole based Schiff bases **5a-b** synthesized by the reaction of pyrrolo[3,2-*c*]carbazole carbohydrazide **4** with benzaldehyde and p-bromobenzaldehyde. The synthetic route to pyrrolo[3,2-*c*]carbazole-2-carboxylate *via* a Hemestberger indole synthesis method has been reported by us [8]. Accordingly, the ethyl ester substituted pyrrolo[3,2-*c*]carbazole **3** has been synthesized by the sequential reaction of 9-ethyl-9*H*-carbazole-3-carbaldehyde **1** with ethyl azidoacetate in the presence of sodium methoxide in ethanol to form the corresponding azido ester **2** as an intermediate. The thermal cyclization of the intermediate **2** was yielded new pyrrolo[3,2-*c*]carbazole **3.** The cyclisation reaction occurred selectively at the C4 position of the carbazole ring rather than the C2 position. Pyrrolo[3,2-*c*]carbazole **3** was synthesized as the starting motif for the construction of targeted Schiff bases. The structure of pyrrolo[3,2-*c*]carbazole **3** was confirmed by spectral data. The most important feature in the ^1^H NMR spectrum of the compound **3** was the appearance of a singlet at 9.34 ppm corresponding to the pyrrole NH proton. In addition, analysis of the ^13^C NMR spectrum revealed that the CH_3_ and CH_2_ carbons of the *N*-ethyl and *O*-ethyl groups gave signals at 9.45, 9.80, 33.27, and 56.07 ppm, respectively. Further evidence for the reaction was provided by the MALDI-TOF mass spectrum, which exhibited a peak at m/z 306, consistent with the proposed structure. Suitable crystal of pyrrolo[3,2-*c*]carbazole **3** was obtained by slow crystallization from a dichloromethane/n-hexane mixture, allowing the determination of its X-ray crystal structure. The compound crystallizes in the monoclinic crystal system (space group *P*2₁/*c*) with unit cell parameters *a* = 15.4257(15) Å, *b* = 4.8587(5) Å, *c* = 20.832(2) Å, β = 99.060(2)°, and *V* = 1541.8(3) Å³ (Z = 4). The structure was refined to satisfactory agreement factors (*R*1 = 0.0420, w*R*2 = 0.0989 for *I* ≥ 2σ(*I*)), confirming the reliability of the model. The cyclization reaction of the vinyl azide intermediate **2** could potentially lead to the formation of two different isomers. The X-ray analysis of the obtained crystals confirmed that the intramolecular cyclization of the intermediate **2** occurred at the C4 position of the carbazole ring, unambiguously establishing the formation of pyrrolo[3,2-*c*]carbazole **3** (Fig. 1). This structural assignment is further supported by key bond lengths within the fused system, which fall within the expected ranges for a conjugated aromatic framework (C–C ≈ 1.36–1.42 Å; C–*N* ≈ 1.36–1.39 Å). The presence of a carbonyl group is confirmed by the short C = O bond [1.204(2) Å], while the overall bond length distribution indicates significant π-delocalization and a nearly planar molecular structure (Scheme 1).

Pyrrolo[3,2-*c*]carbazole-2-carbohydrazide **4**, employed as the amine component in the Schiff base reaction, was synthesized by pyrrolo[3,2-*c*]carbazole **3** with hydrazine hydrazine hydrate in ethanol under reflux, affording the desired carbohydrazide **4** in good yield [21]. With the pyrrolo[3,2-*c*]carbazole-2-carbohydrazide **4** in hand, attention subsequently turned to the synthesis of new Schiff bases **5a-b **(Scheme 1). Condensation of pyrrolo[3,2-*c*]carbazole-2-carbohydrazide **4** with benzaldehyde in ethanol gave the corresponding Schiff base **5a** in 88% yield. Similarly, *p-*bromobenzaldehde was also employed to generate the compounds **5b** under the same reaction conditions. The spectral data, including ^1^H NMR, ^13^C NMR, FT-IR, and mass spectrometry of the targeted Schiff bases **5a-b** were in full agreement with the proposed structures, confirming their successful synthesis (see Supporting Information). The ^1^H NMR and ^13^C NMR spectra of compound **5b** were characteristic for compounds **5a-b**. The ^1^H NMR spectrum of compound **5b** showed the presence of two brought singlets at 11.92 and 12.01 ppm, corresponding to the pyrrole and amide NH protons, respectively. The spectrum also displayed a new imine proton signal at 8.99 ppm. In addition, signals corresponding to *N*-ethyl carbazole moiety were observed, with the methylene (CH_2_) protons appearing as a quartet at 4.55 ppm and the methyl (CH_3_) protons as a triplet at 1.37 ppm. Similarly, methylene (CH_2_) and methyl (CH_3_) appeared at 37.26 and 13.99 ppm in ^13^C NMR spectrum, respectively.

### Photophysical Studies

#### Investigation of the Photophysical Properties of Schiff Base Products 5a and 5b

**Fig. 2:**
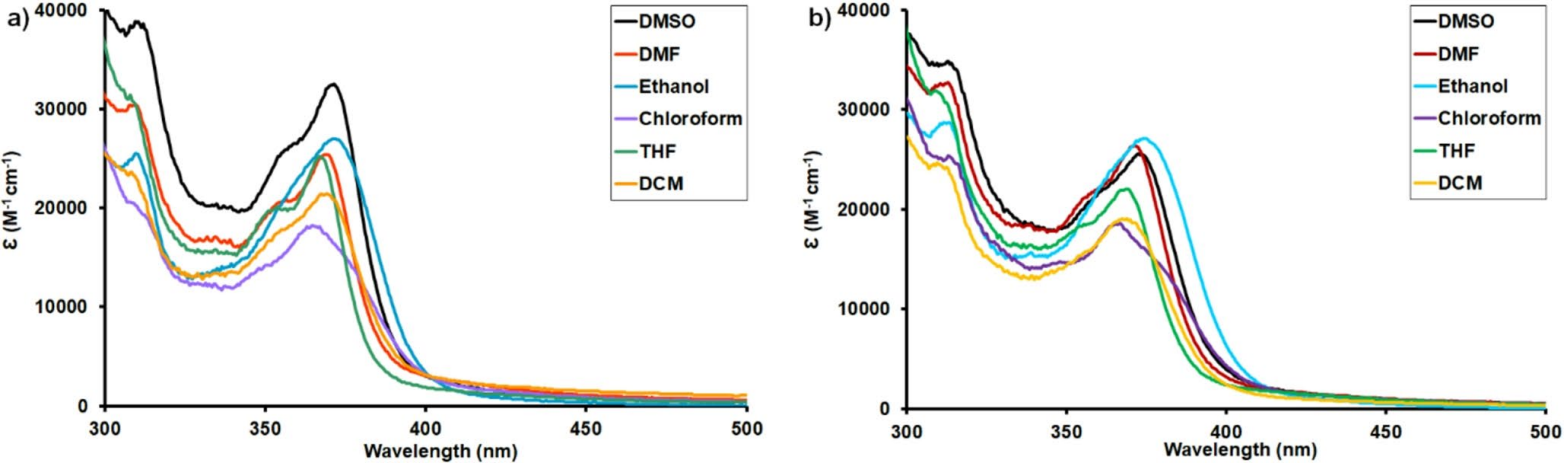
UV–Vis absorption spectra of (**a**) compound **5a** and (**b**) compound **5b** in different solvents

The photophysical properties of the newly synthesized and characterized pyrrolo[3,2-*c*]carbazole-based Schiff bases (**5a** and **5b**) were investigated using UV-Vis and fluorescence spectroscopy. For this purpose, the UV-Vis absorption spectra of compounds **5a** and **5b** were recorded at a concentration of 10 *µ*M in various solvents, including DMSO, DMF, Ethanol, chloroform, THF and DCM at room temperature. The solvent-dependent absorption properties of compounds **5a** and **5b** were thoroughly examined using a series of solvents with different polarities. It was found that the maximum absorption wavelengths remained minimal effect across the different solvent environments. All absorption spectra of compounds **5a** and **5b** revealed slight bathochromic shifts (1–7 nm) in polar solvents such as DMSO, DMF, and ethanol compared with the less polar solvents. In all studied solvents, absorption bands were observed in the range of 300–420 nm, which can be assigned to π→π* transitions of the aromatic conjugated system and n→π* transitions arising from the nonbonding electrons of nitrogen in the imine (–CH = N–) group (Fig. 2).

**Fig. 3:**
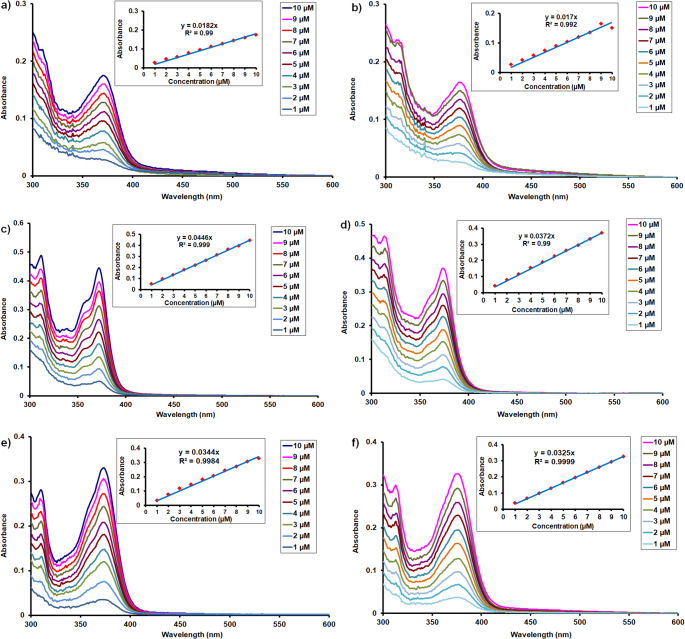
UV–Vis spectra of compounds **5a** and **5b** at varying concentrations in (**a**, **b**) chloroform, (**c**, **d**) DMSO and ethanol (**e**, **f**)

Furthermore, the ground-state absorption spectra of compounds **5a** and **5b** were investigated in chloroform, DMSO and ethanol over a range of concentrations to evaluate possible concentration-dependent effects. As shown in Fig. 3, the proportional change in absorbance was observed with increasing concentration consistent with the Beer–Lambert law. Molar extinction coefficients (ε) of the compounds **5a** and **5b** were calculated as 18,200 and 18,600 in chloroform, 32,400 and 25,600 in DMSO, 27,000 and 27,100 in ethanol respectively. Notably, no shift in the absorption maximum was observed across the investigated concentration range (1–10*µ*M). This concentration-independent absorption wavelength indicates that the electronic transition of the studied compounds remains unchanged and suggests the absence of significant intermolecular interactions or aggregation effects in this solvents under experimental conditions.

In addition, fluorescence emission spectra of both compounds were measured under the same concentration and solvent conditions. The fluorescence spectra, recorded in the 300–700 nm range, did not exhibit a mirror-image relationship with the corresponding absorption spectra, suggesting significant geometric rearrangements upon excitation. This result is further supported by the large Stokes shift values observed for both compounds.

**Fig. 4:**
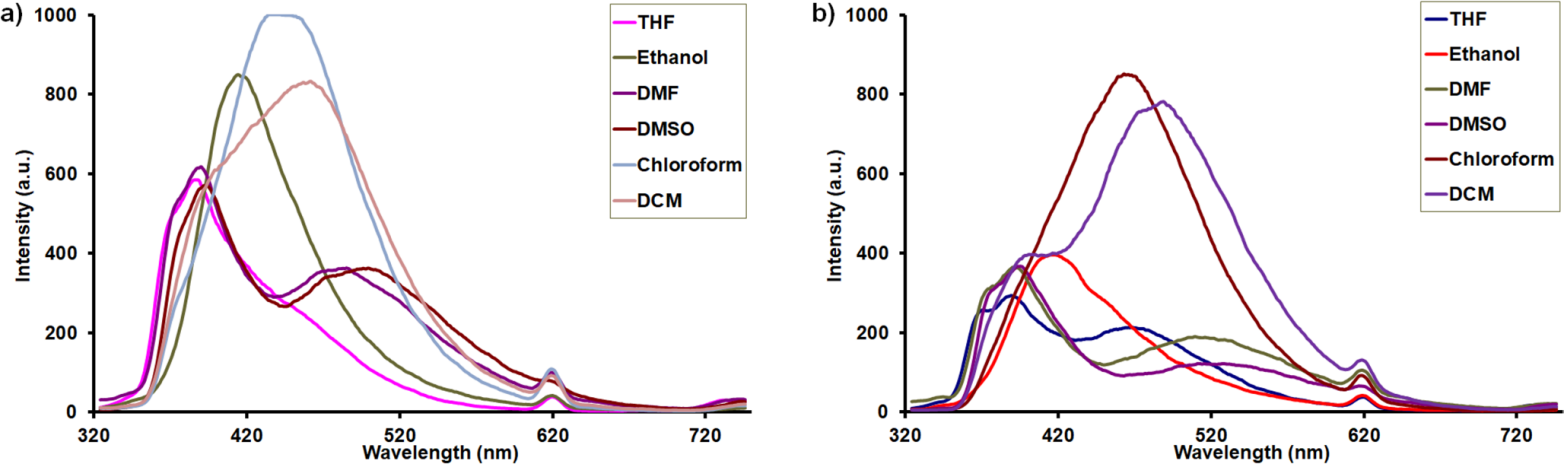
Florescence spectra of **5a** (**a**) and **5b** (**b**) at the concentration 10 *µ*M (λ_ex_ = 310 nm)

**Scheme 2:**
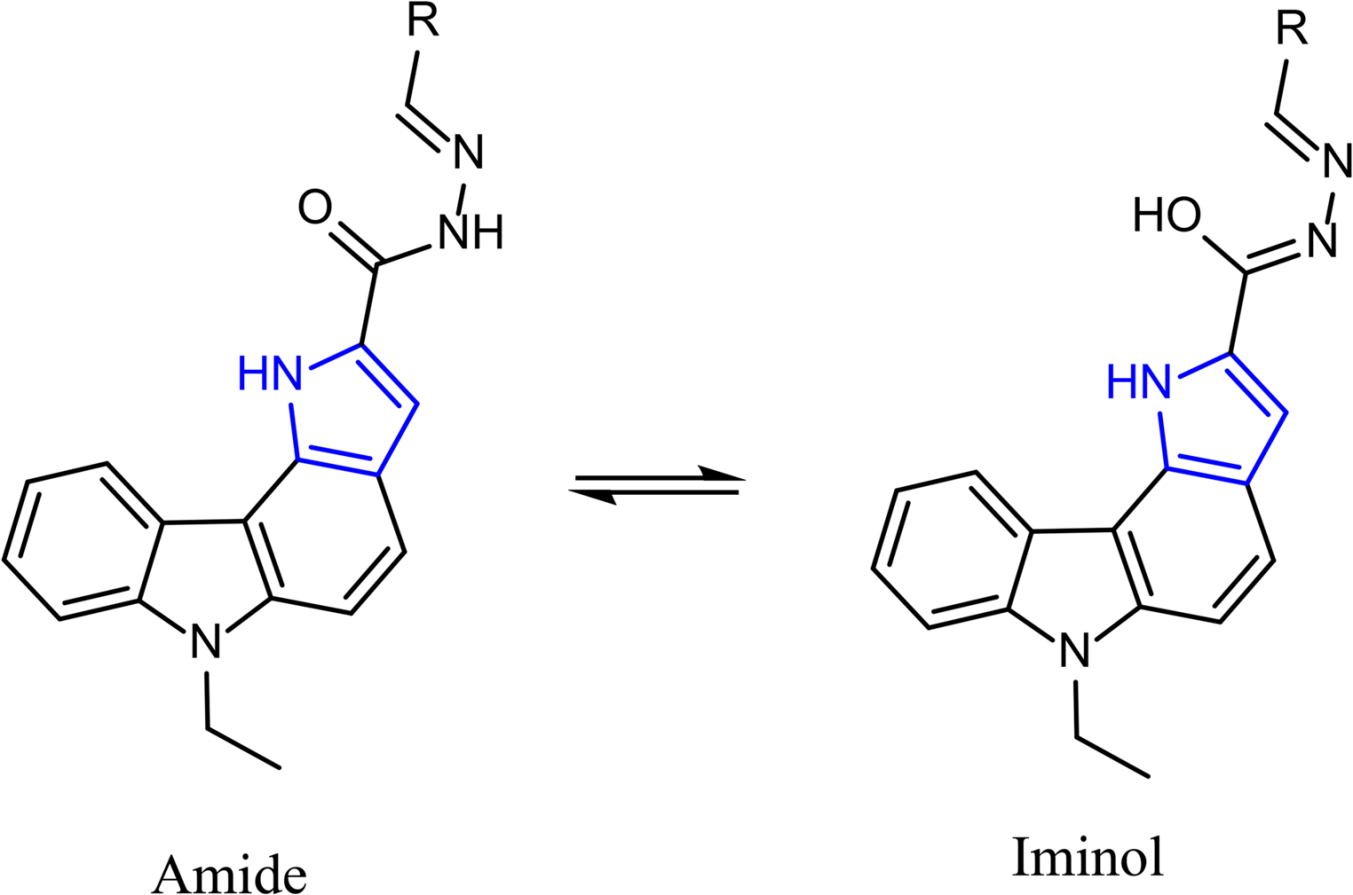
The possible tautomeric forms of amides and their iminol forms

The studied compounds (**5a** and **5b**) exhibit dual emission bands in different solvents, suggesting the occurrence of excited-state proton transfer (Fig. 4, Scheme 2). This dual emission indicates the coexistence of amide-iminol tautomers in the excited state. In these pyrrolo[3,2-*c*]carbazole Schiff bases **5a** and **5b**, the –NH group of the pyrrole ring can act as a proton donor, while the carbonyl (–C = O) and imine (–CH = N–) groups can function as proton acceptors, potentially facilitating the ESIPT process. Excited-state intramolecular proton transfer (ESIPT) is a photophysical process in which a proton migrates within a molecule after excitation, typically between a donor (–OH or –NH) and an acceptor (C = O, imine, or heteroatom) group [20]. In polar solvents such as ethanol, DMF, and DMSO, an iminol emission band was observed along with a comparatively less intensity amide emission peak suggests the operation of an ESIPT mechanism [21–24]. In related carbohydrazide scaffold, the s-trans conformer has been identified as the energetically preferred structure, where its enhanced stability is attributed to conjugation and hydrogen-bonding effects, which in turn play a significant role in determining their conformational and photophysical properties [25]. The fluorescence quantum yield (Φ_F_) value of **5a** and **5b** were determined by comparing with the standard of quinine sulfate in 0.1 M H_2_SO_4_ solution using Eq. (1) and these values were found as 0.44 for **5a** and 0.47 for **5b**. The studied compounds (**5a** and **5b**) showed higher Φ_F_ value in chloroform when compared to di-carbazole based Schiff-base (0.24 in THF) and di-anthracene based Schiff-base (0.03 in THF) groups [26]. Indole-3-carbaldehyde Schiff base derivatives exhibited lower or similiar fluorescence quantum yield (Φ_F_ = 0.07–0.46) compared to the compounds investigated in this study (**5a** and **5b**) in chloroform [27]. The Φ_F_ values of 4-((9 H-carbazol-9-yl)methyl)-N′-(4-methylbenzylidene)benzohydrazide (Φ_F_ = 0.601) and 4-((9 H-carbazol-9-yl)methyl)-N′-(2-hydroxybenzylidene)benzohydrazide (Φ_F_ = 0.703) were found higher the value of the compound **5a** and **5b** [28].

**Fig. 5:**
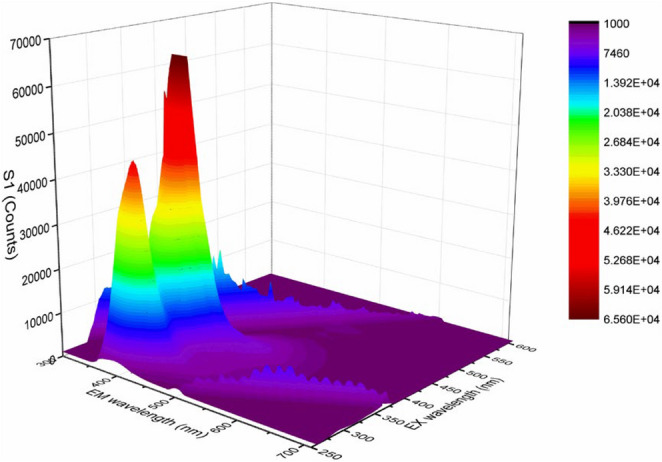
3D fluorescence spectra of **5a** in chloroform

**Fig. 6:**
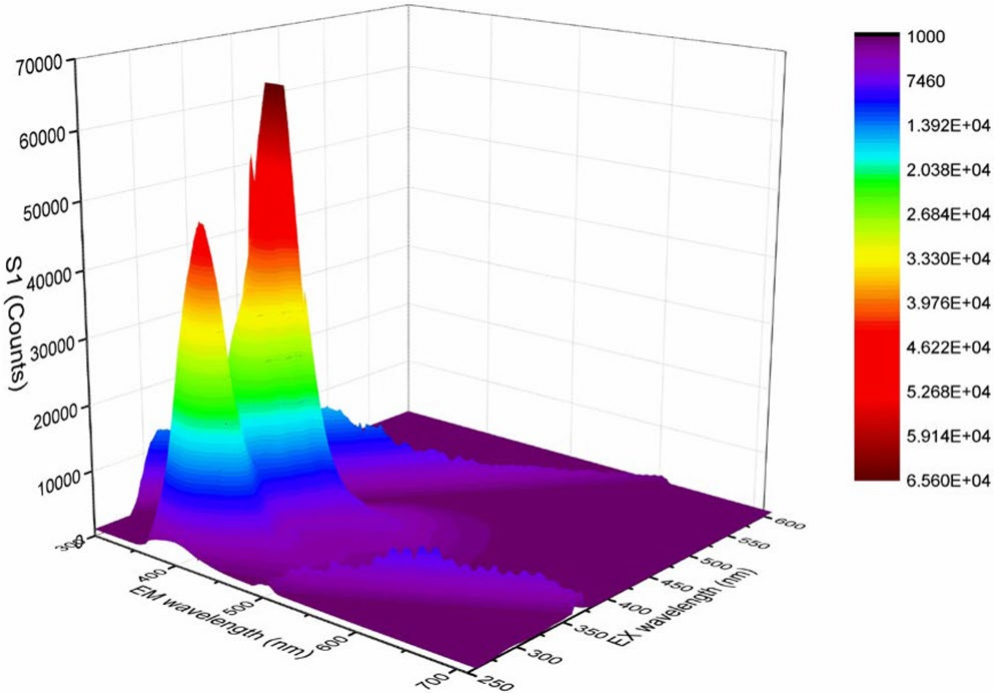
3D fluorescence spectra of **5b** in chloroform

Three-dimensional fluorescence spectroscopy provides detailed information about the fluorescence properties of compounds by simultaneously varying the excitation and emission wavelengths. The 3D fluorescence maps of compounds **5a** and **5b** in chloroform showing their emission peaks in the 300–700 nm range, are presented in Figs. 5 and 6.

**Fig. 7:**
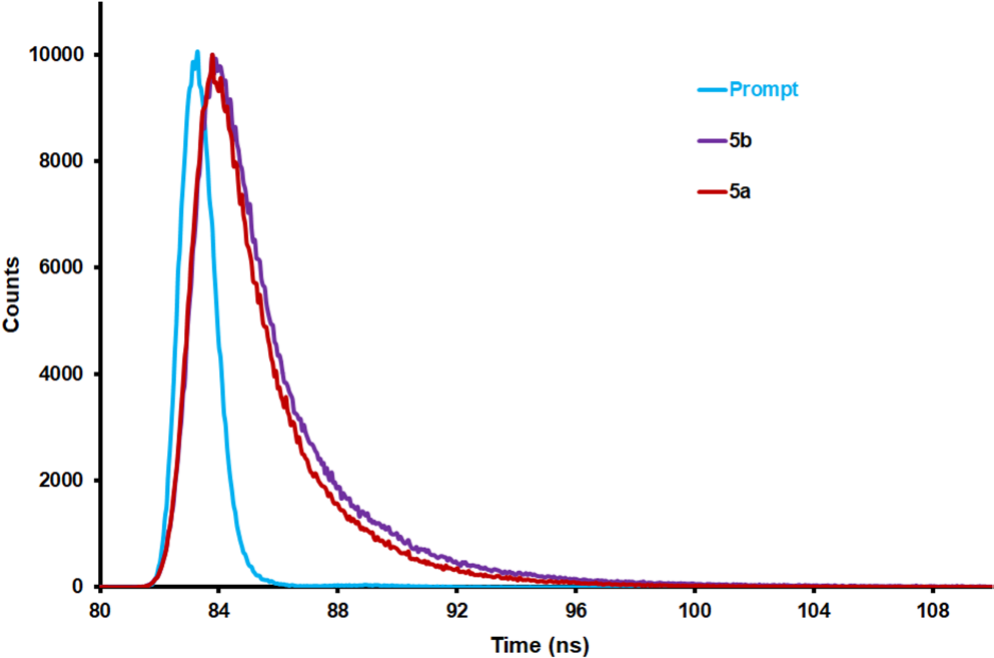
Time correlated single photon counting (TCSPC) fluorescence decay curve of **5a** and **5b** in chloroform

The fluorescence lifetime (τ_F_) profiles of compounds **5a** and **5b** in chloroform are presented in Fig. 7. Accordingly, the fluorescence lifetime of compound **5b** was determined as 1.92 ns (CHISQ = 1.4) in chloroform. Similarly, the fluorescence lifetime of compound **5a** was found to be 1.59 ns (CHISQ = 1.1) in chloroform. The photophysical properties of compounds **5a** and **5b**, such as the fluorescence quantum yield (Φ_F_), fluorescence lifetimes (τ_F_) and molar absorptivities (ε), are tabulated in Tables 1 and 2. Using the measured fluorescence quantum yields (Φ_F_) and fluorescence lifetimes (τ_F_), the radiative decay rate constants (k_f_ = Φ_F_/τ_F_) were calculated, and the obtained k_f_ values are listed in Table 2. The calculated k_f_ values for the two compounds (0.28 for **5a** and 0.25 for **5b**) are very close to each other, indicating that the radiative emission processes are nearly identical.

**Table 1:** Summary of the UV-Vis absorption bands for compound **5a** and **5b**

Solvent	Polarity Index	λ_abs_ (nm, max)	Ɛ (L mol^-1^ cm^-1^) x10^3^	
			5a	5b
DMSO	7.2	310 ~ 370	38.8 32.4	34.9 25.6
DMF	6.4	310 ~ 370	30.4 25.4	32.7 26.4
Ethanol	5.2	310 ~ 370	25.4 27.0	28.7 27.1
Chloroform	4.1	310 ~ 370	20.2 18.2	25.4 18.6
THF	4.0	310 ~ 370	30.7 25.2	30.7 22.0
DCM	3.1	310 ~ 370	23,6 21.4	24,3 19.1

**Table 2:** Photophysical properties of compound **5a** and **5b** in chloroform

	λ_abs_ (nm, max)	λ_em_ (nm, max)	Δ_Stokes_ (nm)	Φ_F_	τ_F_	k_f_ 10^9^s^-1^
5a	365	443	78	0,44	1,59	0.28
5b	367	465	98	0,47	1,92	0.25

## Experimental Section

### Materials and Methods

All reagents and solvents were obtained from commercial sources and appropriately purified, if necessary. The deuterated solvents (deutero chloroform (CDCl_3_) and (deutero dimethyl sulfoxide, (CD_3_)_2_SO, DMSO-d_6_) for NMR spectroscopy were purchased from Merck. IR spectra were recorded between 4000 and 650 cm^− 1^ using a Perkin Elmer Spectrum 100 FT-IR spectrometer. ^1^H and ^13^C NMR spectra were recorded in DMSO-d_6_ solution at 298 K on a Varian 500 MHz spectroscopy. Matrix-assisted laser desorption/ionization time-of-flight mass spectrometry (MALDI-TOF-MS) measurements were performed on a Bruker Daltonics microTOF.

### Measurements

Absorption spectra in the UV-visible region were recorded with a Shimadzu 2101 UV spectroscopy. The dilution of stock solutions prepared with dichloromethane were prepared removing DCM from aliquot for various solvents. Fluorescence measurements were carried out using a Varian Eclipse spectrofluorometer. Slit widths were set to 5 nm and pathlength was 1 cm for all fluorescence experiments. Fluorescence lifetimes (τ_F_) were measured by a Time Correlated Single Photon Counting (TCSPC) method using FLUOROLOG-3 spectrofluorometer, Horiba FL3-2IHR equipped with a NanoLED 310-LH and a standard air cooled R928 PMT detector. A non-fluorescence suspension of colloidal silica (LUDOX 30%, Sigma Aldrich) in water was used as a standard for fluorescence lifetimes measurement. The quality of the fit was judged by the chi-square (χ^2^) values.

### Calculation of Photophysical Parameters

Fluorescence quantum yield (Φ_F_) was determined by the comparative method using (Eq. 1) [28].

1 $$\Phi_F=\Phi_{F\left(Std\right)}\;\frac{F\cdot A_{Std}\cdot\eta^2}{F_{Std}\cdot A\cdot\eta_{Std}^2}$$

where F and F_Std_ are the areas under the fluorescence emission curves of the samples (**5a** and **5b**) and the standard, respectively. A and A_Std_ are the relative absorbance of the samples (**5a** and **5b**) and standard at the excitation wavelength, respectively. ɳ^2^ and ɳ^2^_std_ are the refractive indices of solvents used for the samples and standard, respectively. In this study, quinine sulfate which has Φ_F_ = 0.54 in 0.1 M H_2_SO_4_ was used as a standard [29]. Both the sample and standard were excited at the same wavelength (310 nm).

#### 6-ethyl-1,6-dihydropyrrolo[3,2-*c*] carbazole-2-carboxylate (3)

A solution of sodium methoxide was prepared *via* the portion-wise addition of metallic sodium (0.5 g, 21.73 mmol) to pre-cooled anhydrous ethanol (20 mL) with stirring under nitrogen. The methoxide solution was stirred and cooled to -15 °C in an ice-salt slurry before the dropwise addition of a solution containing carbazole-3-carbaldehyde **1** (0.4 g, 1.86 mmol) and methyl azidoacetate (2 g, 17.39 mmol) in anhydrous THF (6 mL) over 1 h, under nitrogen atmosphere. The mixture was stirred further for 4 h with cooling and then poured onto crushed ice. This reaction gave the vinyl ester intermediate, which was not isolated, but rather underwent cyclisation in 1,2-dichlorobenzene (10 mL) and the resulting mixture was refluxed for 3 h. The solvent was then distilled under reduced pressure and the residue was purified by flash column chromatography using dichloromethane/hexane (8:2) as eluent to give title compound as a yellow solid; yield as a yellow solid (0.57 g, %68). IR (KBr): ʋ_max 3336, 1690, 1638, 1536, 1493, 1342, 1307, 1256, 1200, 1178, 1148 cm^− 1^; ^1^H NMR (500 MHz, CDCl_3_): δ 9.37 (s, 1H), 8.20 (d, J = 7.8 Hz, 1H), 7.77 (d, J = 8.7 Hz, 1H), 7.58–7.47 (m, 2 H), 7.45 (d, J = 2.0 Hz, 1H), 7.42–7.32 (m,2 H), 4.54–4.44 (m, 4 H), 1.69 (s), 1.49 (m, 6 H) ppm; ^13^C NMR (126 MHz, CDCl_3_) δ 157.4, 134.0, 133.7, 127.3, 120.5, 119.5, 116.5, 115.9, 114.7, 105.6, 104.1, 101.6, 100.1, 56.0, 33.2, 9.8, 9.45 ppm; MALDI-TOF; (C_19_H_18_N_2_O_2_) [M]^+^ calculated: 306.13, found: 306.06.

#### 6-ethyl-1,6-dihydropyrrolo[3,2-*c*]carbazole-2-carbohydrazide (4)

To a stirred suspension of compound **3** (0.40 g, 1.4 mmol) in ethanol (17 mL) hydrazine hydrate (2.0 ml, 41 mmol) was added. Brown color reaction mixture was stirred under reflux for 20 h. The resulting reaction mixture was then cooled to room temperature and filtered to afford the compound **4** (0.24 g, 60%) as yellow solid ^1^H NMR (500 MHz, DMSO-d_6_): δ 11.87 (s, 1H), 9.91 (s, 1H), 9.00 (d, J = 7.8 Hz, 1H), 7.69 (d, J = 8.7 Hz, 1H), 7.62 (d, J = 8.2 Hz, 1H), 7.44–7.38 (m, 2 H), 7.29 (d, J = 1.9 Hz, 1H), 7.24 (t, J = 7.4 Hz, 1H), 4.50 (q, J = 7.1 Hz, 2 H), 1.32 (t, J = 7.1 Hz, 3 H) ppm; ^13^C NMR (126 MHz, DMSO-d_6_): δ 161.0, 138.0, 137.7, 130.6, 128.8, 123.9, 122.3, 120.7, 120.6, 119.9, 118.6, 108.7, 106.5, 106.2, 104.2, 37.2, 14.0 ppm; FT-IR: ʋ_max 3296, 2970, 1634, 1538, 1323, 1150 cm^− 1^; MALDI-TOF; C_17_H_16_N_4_O [M + H]^+^ calculated: 292.13; found [M-H]^+^: 291.10.

#### N-(benzylidene)-6-ethyl-1 H-pyrrolo[3,2-*c*]carbazole-2-carbohydrazide (5a)

A mixture of compound **4** (100 mg, 0.34 mmol) and benzaldehyde (37 mg, 0.34 mmol) was stirred in methanol (5 mL) at room temperature, followed by the addition of two drops of acetic acid. The reaction mixture was refluxed with continuous stirring for 3.5 h. Upon completion, the resulting brown suspension was filtered to afford the desired product **5a** as a brown solid in 97 mg yield (88%). ^1^H NMR (500 MHz, DMSO-d_6_) δ 11.99 (bs, 1H), 11.85 (bs, 1H), 8.98 (d, *J* = 6.9 Hz, 1H), 8.48 (s, 1H), 7.78 (d, *J* = 7.5 Hz, 3 H), 7.66 (d, *J* = 8.2 Hz, 1H), 7.58–7.41 (m, 6 H), 7.29–7.24 (m, 1H), 4.53 (q, *J* = 7.1 Hz, 2 H), 1.35 (t, *J* = 7.1 Hz, 3 H) ppm; ^13^C NMR (125 MHz, d-DMSO) δ 161.38, 138.00, 137.96, 134.51, 131.20, 129.87, 128.87, 128.24, 126.99, 124.01, 122.26, 120.80, 120.26, 118.71, 108.81, 106.37, 104.75, 37.28, 14.01 ppm; FT-IR: ʋ_max 3251, 3051, 2963, 1634, 1603, 1509, 1467, 1308, 1249, 1168, 1170, 1025, 967, 827 cm^− 1^; MALDI-TOF; C_24_H_20_N_4_O [M]^+^ calculated: 380.16; found [M]^+^: 380.06.

#### N-(4-bromobenzylidene)-6-ethyl-1 H-pyrrolo[3,2-*c*]carbazole-2-carbohydrazide (5b)

A mixture of compound **4** (100 mg, 0.34 mmol) and p-bromobenzaldehyde (61 mg, 0.34 mmol) was stirred in methanol (5 mL) at room temperature, followed by the addition of two drops of acetic acid. The reaction mixture was refluxed with continuous stirring for 3.5 h. The resulting yellow suspension was filtered, and the crude product was purified by column chromatography (dichloromethane: ethyl acetate, 30:70) to afford the target compound **5b** as a yellow solid in 82 mg yield (53%). ^1^H NMR (500 MHz, DMSO-d_6_) δ 12.01 (bs, 1H), 11.92 (bs, 1H), 9.00 (d, *J* = 7.6 Hz, 1H), 8.46 (s, 1H), 7.80 (d, *J* = 8.7 Hz, 1H), 7.77–7.73 (m, 2 H), 7.73–7.65 (m, 3 H), 7.61–7.53 (m, 1H), 7.50 (d, *J* = 8.8 Hz, 1H), 7.48–7.43 (m, 1H), 7.31–7.24 (m, 1H), 4.55 (q, *J* = 7.0 Hz, 2 H), 1.37 (t, *J* = 7.1 Hz, 3 H) ppm; ^13^C NMR (125 MHz, DMSO-d_6_) δ 163.07, 138.00, 137.94, 133.81, 131.84, 131.24, 128.83, 124.00, 122.99, 122.35, 120.79, 120.26, 118.70, 108.79, 106.35, 104.77, 37.26, 13.99 ppm; FT-IR: ʋ_max 3449, 3255, 2973, 1682, 1604, 1590, 1490, 1343, 1262, 1066, 1008, 820, 742 cm-1; MALDI-TOF; C_24_H_19_BrN_4_O [M]^+^ calculated: 458.07; found [M]^+^: 458.27.

## Conclusion

In this study, novel pyrrolo[3,2-*c*]carbazole-based Schiff bases **5a** and **5b** were successfully synthesized and fully characterized. UV-Vis absorption spectra showed minimal solvent- and concentration-dependent effects, indicating stable ground-state electronic properties. Fluorescence investigations revealed dual emission bands, which may be associated with amide–iminol tautomerism, with possible contributions from excited state processes in polar solvents. The compounds exhibited notable fluorescence quantum yields and well-defined absorption/emission characteristics. The results provide insight into photophysical behavior of pyrrolo[3,2-*c*]carbazole Schiff bases.

## Supplementary Information

Below is the link to the electronic supplementary material.

Supplementary Material 1 (DOCX 487 KB)

## Data Availability

No datasets were generated or analysed during the current study.
