# Supplementary material for: Design and Photophysical Investigation of Schiff Base-Functionalized Pyrrolo[3,2-c]carbazoles
Source: J Fluoresc. 2026 May 14;36(5):3623–32. doi: 10.1007/s10895-026-04792-7 (PMC13226360; doi:10.1007/s10895-026-04792-7)
Supplement: Supplementary file 1 — Supplementary Material 1 (DOCX 487 KB) [file 10895_2026_4792_MOESM1_ESM.docx]

**Design and Photophysical Investigation of Schiff Base-Functionalized Pyrrolo[3,2-c]carbazoles**

Gökhan Özbek,^1^ Esra Nur Kaya,^1^ Mehmet F. Saglam^1^, Hakan Kandemir^2^*,* Ibrahim F. Sengul^1^*^*^*

^1^Department of Chemistry, Faculty of Science, Gebze Technical University, Gebze, Kocaeli, Türkiye

^2^Department of Chemistry, Faculty of Art and Science, Tekirdag Namık Kemal University, Tekirdag, Türkiye

*Correspondences: [fazilsengul@gtu.edu.tr](mailto:fazilsengul@gtu.edu.tr), enkaya@gtu.edu.tr


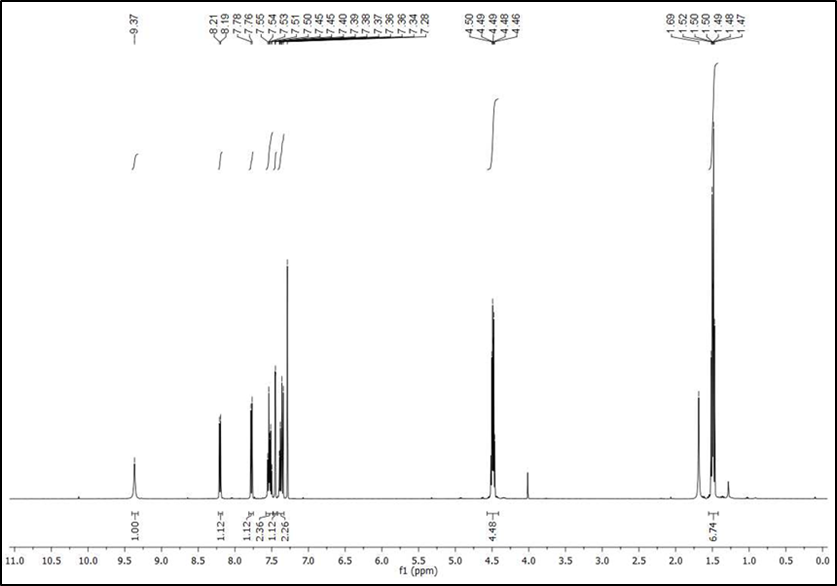


**Figure S1.** ^1^H NMR spectra of compound **3**


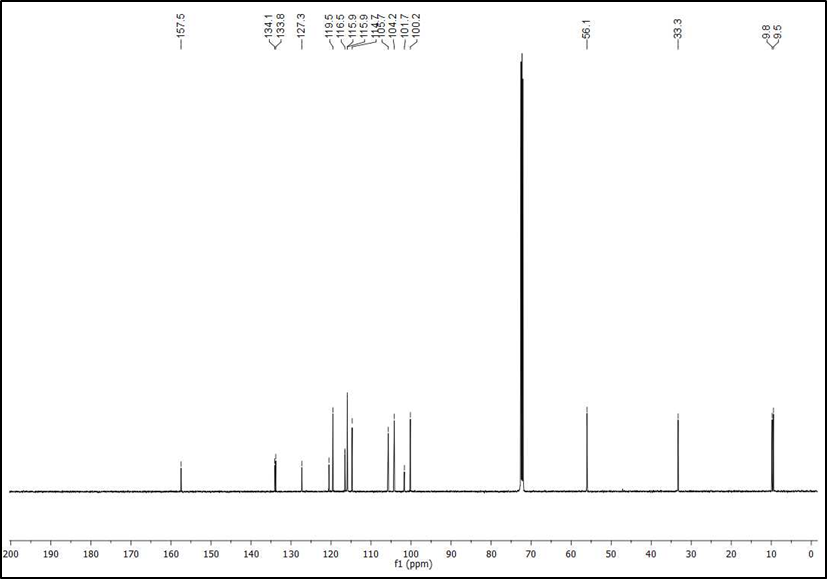


**Figure S2.** ^13^C NMR spectra of compound **3**


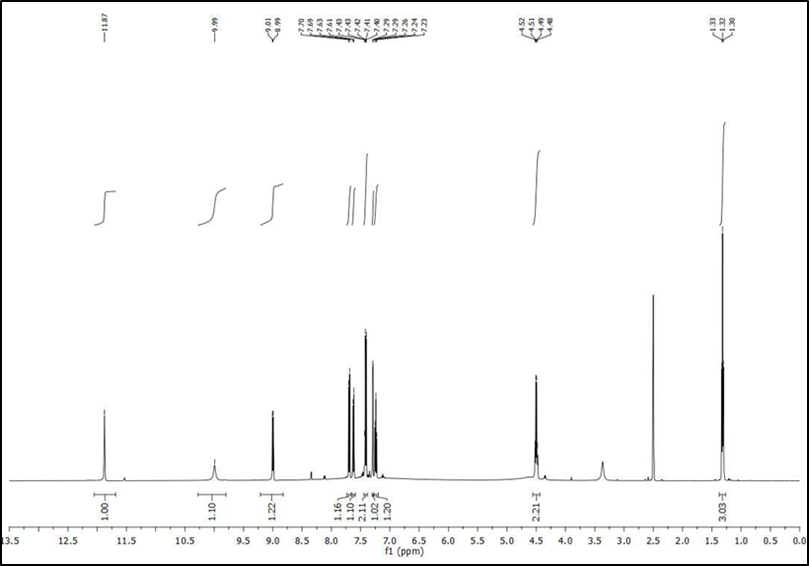


**Figure S3.** ^1^H NMR spectra of compound **4**


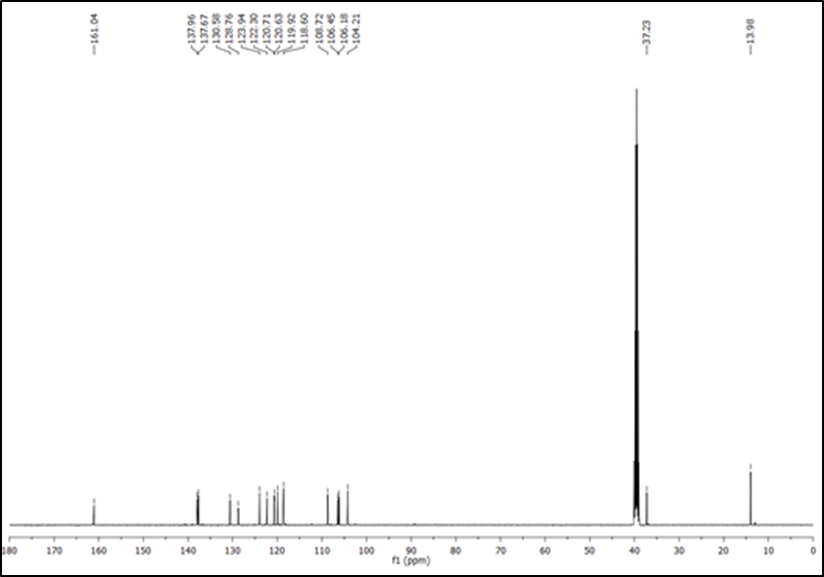


**Figure S4.** ^13^C NMR spectra of compound **4**


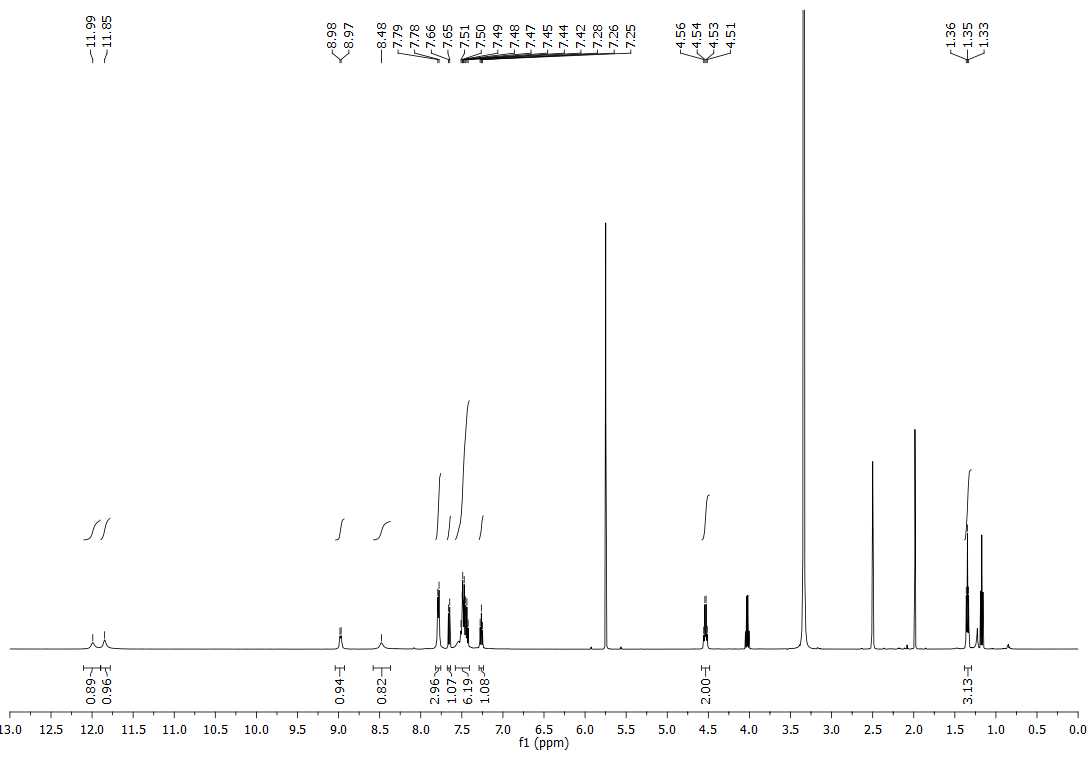


**Figure S5.** ^1^H NMR spectra of compound **5a**


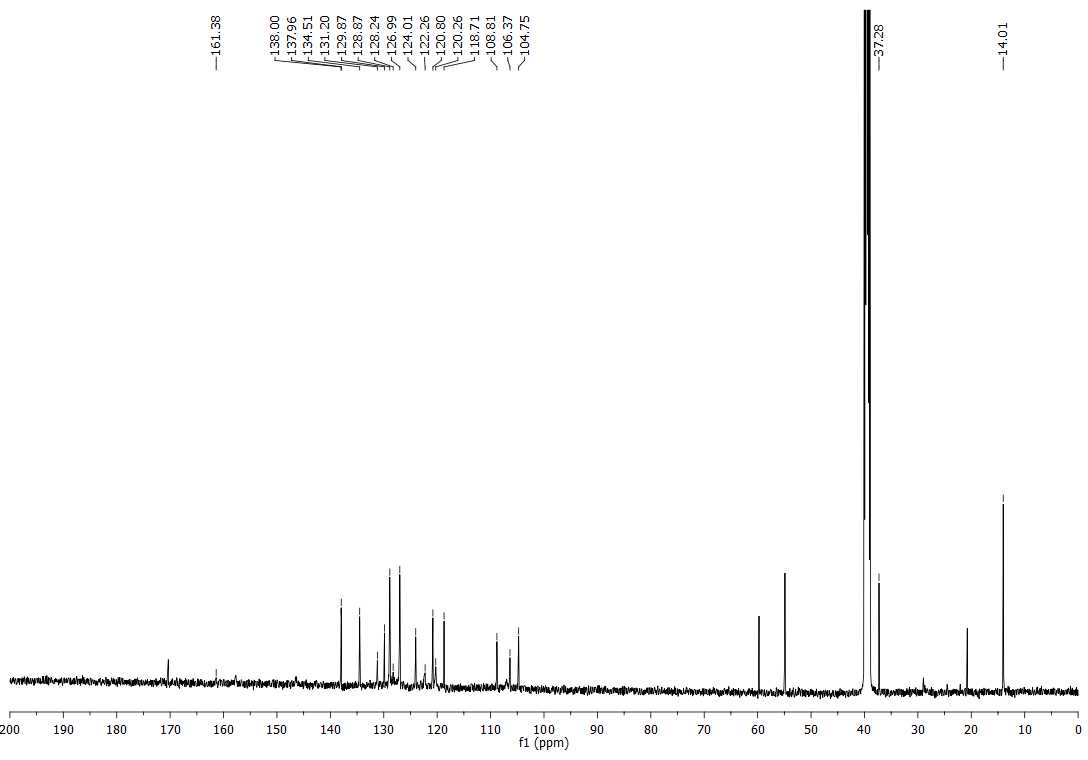


**Figure S6.** ^13^C NMR spectra of compound **5a**


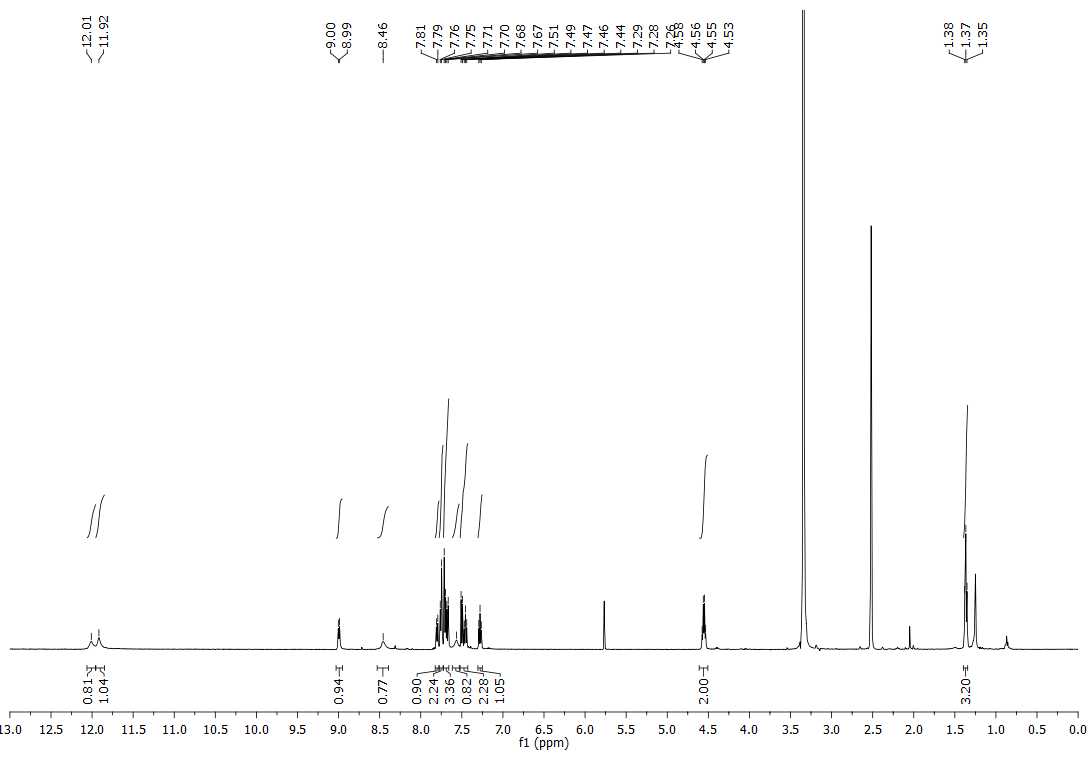


**Figure S7.** ^1^H NMR spectra of compound **5b**


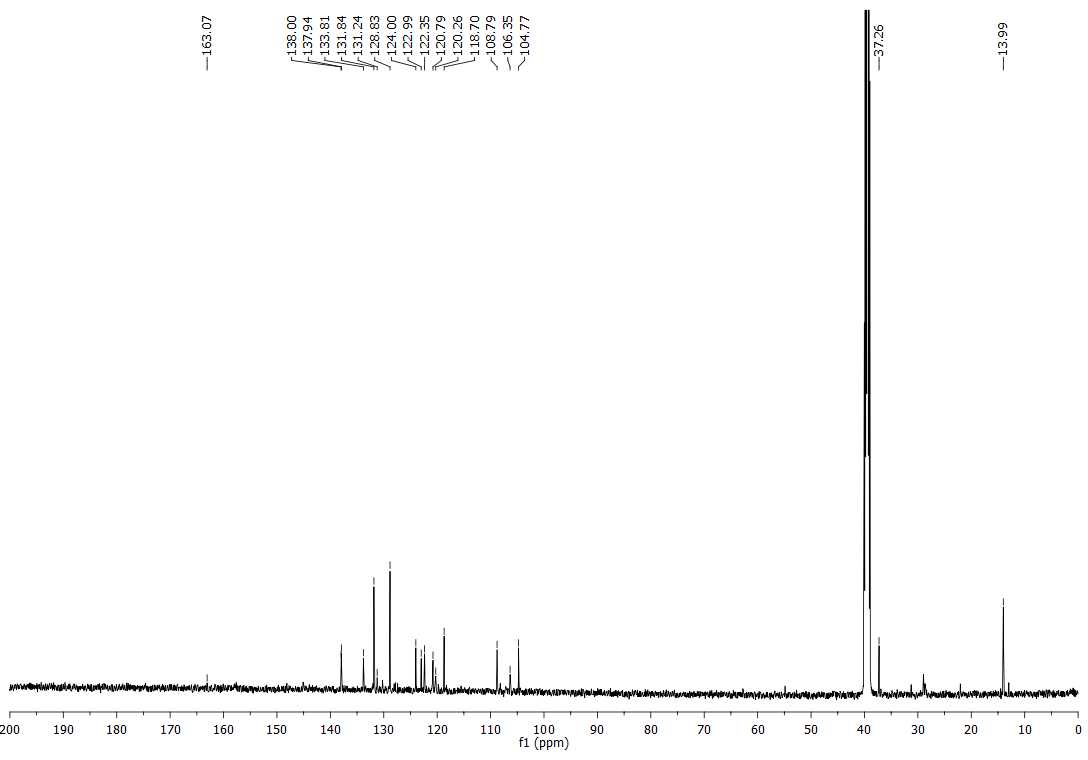


**Figure S8.** ^13^C NMR spectra of compound **5b**

| **Identification code** | Compound **4** |
| --- | --- |
| **Empirical formula** | C_19_H_18_N_2_O_2_ |
| **Formula weight** | 306.35 |
| **Temperature/K** | 273.15 |
| **Crystal system** | monoclinic |
| **Space group** | P2_1_/c |
| **a/Å** | 15.4257(15) |
| **b/Å** | 4.8587(5) |
| **c/Å** | 20.832(2) |
| **α/°** | 90 |
| **β/°** | 99.060(2) |
| **γ/°** | 90 |
| **Volume/Å^3^** | 1541.8(3) |
| **Z** | 4 |
| **ρ_calc_g/cm^3^** | 1.320 |
| **μ/mm^‑1^** | 0.087 |
| **F(000)** | 648.0 |
| **Crystal size/mm^3^** | 0.693 × 0.074 × 0.058 |
| **Radiation** | MoKα (λ = 0.71073) |
| **2Θ range for data collection/°** | 3.96 to 50.078 |
| **Index ranges** | -18 ≤ h ≤ 18, -5 ≤ k ≤ 5, -24 ≤ l ≤ 24 |
| **Reflections collected** | 18640 |
| **Independent reflections** | 2724 [R_int_ = 0.0593, R_sigma_ = 0.0401] |
| **Data/restraints/parameters** | 2724/0/210 |
| **Goodness-of-fit on F^2^** | 1.035 |
| **Final R indexes [I>=2σ (I)]** | R_1_ = 0.0420, wR_2_ = 0.0989 |
| **Final R indexes [all data]** | R_1_ = 0.0644, wR_2_ = 0.1128 |
| **Largest diff. peak/hole / e Å^-3^** | 0.16/-0.16 |

**Table S1.** Crystal data and refinement parameters for compound **4**
